# Switching from IL23 inhibitors to IL17 inhibitors: A safe and effective practice?

**DOI:** 10.1111/dth.15697

**Published:** 2022-07-19

**Authors:** Luca Mastorino, Gabriele Roccuzzo, Paolo Dapavo, Niccolò Siliquini, Gianluca Avallone, Marco Rubatto, Pietro Quaglino, Simone Ribero

**Affiliations:** ^1^ Department of Medical Sciences, Dermatologic Clinic University of Turin Turin Italy

Dear Editor

Psoriasis is an immune‐mediated skin disease characterized by an increased inflammatory response mediated by the IL23/IL17 axis.^1^, ^2^ The latest generation of biologic drugs (i.e., guselkumab, risankziumab, and tildrakizumab) selectively inhibit the p19 subunit of interleukin 23 (IL23),^3^, ^4^, ^5^, ^6^ which acts upstream of the IL23/IL17 pathway. The efficacy of these drugs has been confirmed by phase three studies which showed safety profile and remarkable effects in terms of Psoriasis Area Severity Index (PASI) 90 and 75, achieved by more than 75% of patients treated with guselkumab/Risankizumab and more than 60% of patients treated with Tildrakizumab.^4^, ^5^, ^6^ While these molecules demonstrated superiority over older tumour necrosis factor (TNF) alpha inhibitors, efficacy over anti‐IL17 was not always evident.^4^, ^6^, ^7^ IL23 blockade doesn't always work for psoriasis and can lose efficacy after initial benefit. Herein, we report the experience of our clinic on patients who switched from anti‐IL23 to IL17. In the last year, out of 769 patients with moderate‐ severe psoriasis followed at the Dermatology Clinic of the Turin University Hospital, seven patients switched to an IL17 inhibitor after treatment failure with IL23 inhibitor (Table 1). In all seven cases, the switch was made directly without wash‐out and intercurrent therapy. The mean age was 48.7 years (ds 6.1), with a mean onset of psoriasis at 25 (ds 2.7). The population was mainly female (five out of seven patients). The mean BMI was 25, none of the patients was obese or diabetic, 2 patients had an increased cardiovascular risk. Six patients suffered from psoriasis vulgaris, one patient presented a pustular form. Three patients had joint involvement. No significant differences were found with the general population in our clinic (Table 1). Six patients discontinued guselkumab and one patient discontinued risankizumab. Four out of seven patients had previously used more than one biologic agent. The mean follow‐up of treatment with anti‐IL23 was 7 months, primary ineffectiveness was the first cause of discontinuation (five out of seven cases), followed by secondary ineffectiveness. One case of perimalleolar edema was reported in risankizumab‐treated patients. Four patients subsequently started brodalumab and 3 ixekizumab, one patient performed a rechallenge with ixekizumab. Currently, all patients are continuing these treatments. The initial mean PASI at the switch was 8.3 (ds 2.3) and after 16 weeks dropped to 1.1 (ds 2.0), with five out of seven patients achieving PASI100; in the following weeks the response was maintained with mean PASI at weeks 24, 40 and 52 of 1.4, 1.4, and 1.6, respectively. No side effects were reported.

**TABLE 1:** Patients' characteristics and outcomes, and comparison with the general psoriatic population at our clinic in the time analyzed

No. of patients	Age	Sex	BMI	Age of onset	Type of Psoriasis	PSA	Bilogic in course	Previous biological therapy	PASI at baseline	PASI 16w	PASI 90 16w	PASI100W	PASI a 28settimane	PASI90 28 W	PASI 10028 W	PASI 40 W	PASI90 40 W	PASI 10040 W	PASI 52 W	PASI90 52 W	PASI 10052 W	Follow up (months)	Cause of IL23inhibitors suspension	Obesity	Cardiovascular disease	Diabetes Mellitus
1	62	1	27	46	Vulgar	No	Brodalumab	Ixekizumab, risankizumab	8	0	Yes	Yes	0	Yes	Yes	2	No	No				5	Prior inefficacy, adverseevent	No	Yes	No
2	66	0	25	4	Vulgar	Yes	Brodalumab	Ixekizumab, guselkumab	8	0	No	No	0	Yes	Yes	2	No	No	0	Yes	Yes	5	Second inefficacy	No	No	No
3	51	1	28	26	Vulgar	No	Ixekizumab	Secukinumab, ixekizumab, guslkumab	6	0	Yes	Yes	0	Yes	Yes	0	Yes	Yes	0	Yes	Yes	6	Prior inefficacy,	No	No	No
4	34	0	20	18	Vulgar, inverse	No	Brodalumab	Guselkumab	14	5	No	No	5	No	No	5	No	No	5	No	No	12	Second inefficacy	No	No	No
5	22	0	29	16	Vulgar	No	Brodalumab	Guselkumab	7	0	Yes	Yes	0	Yes	Yes	0	Yes	Yes				6	Prior inefficacy	No	No	No
6	45	0	21	11	Vulgar	Yes	Ixekizumab	Guselkumab	5	0	Yes	Yes	0	Yes	Yes	1	No	No				10	Prior inefficacy	No	No	No
7	61	0	25	55	Pustolar	Yes	Ixekizumab	Ustekinumab, guselkumab	10	3	No	No	3	No	No	0	Yes	Yes				7	Prior inefficacy	No	Yes	No
							Switched patients (7)						General population (783)									*p*‐value				
Sex (F/M)							71.4%						35.5%									0.104				
Age (mean)							48.7						52.2									0.886				
Age of onset (mean)							25.1						34.3									0.947				
PSA (yes/no)							42.8%						28.7%									0.475				
BMI (mean)							25						27									0.225				
Obesity (yes/no)							0%						23.8%									1				
Cardiovascular disease (yes/no)							28.6%						44%									0.475				
Diabetes Mellitus (yes/no)							0%						12.7%									1				

Abbreviation: PASI, Psoriasis severity index.

Switches from one generation of biologics to the previous one has been previously described and few cases from secukinumab to ustekinumab or adalimumab, with subsequent good responses, have been reported.^1^, ^8^ In most of our patients, the reason for switching was an initial failure of therapy, with subsequent initiation of IL17 receptor inhibitor with brodalumab and to a lesser extent ixekizumab. A rapid response was obtained in all patients. The biological reason for these observations remains to be investigated, primarily due to the complex biology of psoriasis. Probably a more selective inhibition of the cytokine cascade in some patients allows a better response or allows evasion of hypothetical antibodies inactivating the anti‐IL23. In the case of rechallenge, the intercurrent use of anti‐IL23 could guarantee a modification of the cytokine milieu and the patient's immune response, allowing a resumption of the efficacy of IL17 inhibitor after initial treatment. Our evidence and our hypotheses must be confirmed by larger sample sizes studies. Nevertheless, the switch to an earlier monoclonal antibody may be an adequate therapeutic option in psoriatic patients who fail an anti‐IL23.

## AUTHOR CONTRIBUTIONS

Luca Mastorino and Gabriele Roccuzzo made the data analysis, write and review the manuscript. Pietro Quaglino and Simone Ribero made the supervision, review and approved the manuscript. Paolo Dapavo and Niccolò Siliquini made the supervision and approved the manuscript. Gianluca Avallone and Marco Rubatto made the visualization, read and approved the manuscript.

## FUNDING INFORMATION

None.

## CONFLICT OF INTEREST

The author declares that there is no conflict of interest.

## DECLARATION

All the patients signed informed written consent.

## Data Availability

Data available upon reasonable request.
